# Other PHAC publications

**DOI:** 10.24095/hpcdp.45.6.07

**Published:** 2025-06

**Researchers from the Public Health Agency of Canada also contribute to work published in other journals and books. Look for the following articles published in 2025:**

**Alami A, Dave S, Ebrahim M, Zareef I, Uhlik C, Laroche J**. Exploring parent-driven determinants of COVID-19 vaccination in Indigenous children: insights from a national survey. Vaccines (Basel). 2025;13(2):132. https://doi.org/10.3390/vaccines13020132

Hamm NC, **Bartholomew S**, Zhao Y, Peterson S, Al-Azazi S, McGrail K, et al. Minimum elements for reporting a multi-jurisdiction feasibility assessment of algorithms based on routinely collected health data: Health Data Research Network Canada recommendations. Int J Popul Data Sci. 2025;10(2):2466. https://doi.org/10.23889/ijpds.v10i2.2466

Ramage K, **Yee J, Srugo S**, Little J, **Liu S**. Prenatal opioid use disorder and the risk of congenital anomalies in offspring: a population-based study. Birth Defects Res. 2025;117(2):e2456. https://doi.org/10.1002/bdr2.2456

Reynolds DL, Daoust R, **Subnath M, Limburg H**. Preventing harms from first prescriptions of opioids. CMAJ. 2025;197(5):E133-4. https://doi.org/10.1503/cmaj.250094

Salman L, Gien LT, Vicus D, Shier M, Kupets R, **Gibbons S, Ashfaq S, Severini A**, et al. What are the current HPV types contributing to cervical high-grade squamous intraepithelial lesions, adenocarcinoma in situ, and early cervical cancer? J Obstet Gynaecol Can. 2025;47(3):102783. https://doi.org/10.1016/j.jogc.2025.102783

**Shi Y, Macrae K, de Groh M, Thompson W**, Stockwell T. Mortality and hospitalizations fully attributable to alcohol use before versus during the COVID-19 pandemic in Canada. CMAJ. 2025;197(4):E87-95. https://doi.org/10.1503/cmaj.241146

**Shields M, Tonmyr L, Pollock NJ**, Gonzalez A, **McKinnon B**, Joshi D, **Crompton L**, et al. Health status among women and men in Canada who reported experiences of non-physical intimate partner violence, Canada. Prev Med Rep. 2025;51:103011. https://doi.org/10.1016/j.pmedr.2025.103011

Vigod SN, Babujee A, Huang A, Fung K, **Vercammen K, Lye J, Dzakpasu S, Luo W**. Perinatal mental illness in Ontario (2007–2021): a population-based repeated cross-sectional surveillance study. Can J Public Health. Epub 2025 Feb 24. https://doi.org/10.17269 /s41997-024-00987-2

Wang J, **Orpana H**, Carrington A, Kephart G, Vasiliadis H-M, Leikin B. Development and validation of prediction models for perceived and unmet mental health needs in the Canadian general population: model-based synthetic estimation study. JMIR Public Health Surveill. 2025;11:e66056.https://doi.org/10.2196/66056

